# Genomic Insight of VIM-harboring IncA Plasmid from a Clinical ST69 *Escherichia coli* Strain in Italy

**DOI:** 10.3390/microorganisms8081232

**Published:** 2020-08-12

**Authors:** Vittoria Mattioni Marchetti, Ibrahim Bitar, Aurora Piazza, Alessandra Mercato, Elena Fogato, Jaroslav Hrabak, Roberta Migliavacca

**Affiliations:** 1 Department of Microbiology, Faculty of Medicine, University Hospital in Pilsen, Charles University, 32300 Pilsen, Czech Republic; vittoria.mattionimarche01@universitadipavia.it (V.M.M.); Jaroslav.Hrabak@lfp.cuni.cz (J.H.); 2 Biomedical Center, Faculty of Medicine in Pilsen, Charles University, 32300 Pilsen, Czech Republic; 3 Department of Clinical-Surgical, Diagnostic and Pediatric Sciences, Unit of Microbiology and Clinical Microbiology, University of Pavia, 27100 Pavia, Italy; aurora.piazza@unipv.it (A.P.); alemercato93@gmail.com (A.M.); roberta.migliavacca@unipv.it (R.M.); 4 Clinical Microbiology Laboratory, ASP Golgi-Redaelli Hospital, Via Olmetto, 20123 Milan, Italy; e.fogato@golgiredaelli.it

**Keywords:** *E. coli*, *bla*VIM-1, *IncA*

## Abstract

*Background*: VIM (Verona Integron-encoded Metallo-beta-lactamase) is a member of the Metallo-Beta-Lactamases (MBLs), and is able to hydrolyze all beta-lactams antibiotics, except for monobactams, and including carbapenems. Here we characterize a VIM-producing IncA plasmid isolated from a clinical ST69 *Escherichia coli* strain from an Italian Long-Term Care Facility (LTCF) inpatient. *Methods*: An antimicrobial susceptibility test and conjugation assay were carried out, and the transferability of the *bla*_VIM-type_ gene was confirmed in the transconjugant. Whole-genome sequencing (WGS) of the strain 550 was performed using the Sequel I platform. Genome assembly was performed using “Microbial Assembly”. Genomic analysis was conducted by uploading the contigs to ResFinder and PlasmidFinder databases. *Results:* Assembly resulted in three complete circular contigs: the chromosome (4,962,700 bp), an IncA plasmid (p550_IncA_VIM_1; 162,608 bp), harboring genes coding for aminoglycoside resistance (*aac(6′)-Ib4*, *ant(3″)-Ia*, *aph(3″)-Ib*, *aph(3′)-XV*, *aph(6)-Id*), beta-lactam resistance (*bla*_SHV-12_, *bla*_VIM-1_), macrolides resistance (*mph(A)*), phenicol resistance (*catB2*), quinolones resistance (*qnrS1*), sulphonamide resistance (*sul1*, *sul2*), and trimethoprim resistance (*dfrA14*), and an IncK/Z plasmid (p550_IncB_O_K_Z; 100,306 bp), free of antibiotic resistance genes. *Conclusions:* The increase in reports of IncA plasmids bearing different antimicrobial resistance genes highlights the overall important role of IncA plasmids in disseminating carbapenemase genes, with a preference for the *bla*_VIM-1_ gene in Italy.

## 1. Introduction

VIM (Verona Integron-encoded Metallo-beta-lactamase) is a member of the Metallo-beta-Lactamases (MBLs), and is able to hydrolyze all beta-lactams antibiotics, except for monobactams, but including carbapenems. The VIM family was first detected and described in Italy from a *Pseudomonas aeruginosa* (*P. aeruginosa*) strain isolated in 1997 [1]; to date, 69 different enzyme variants have been described and grouped in sub-lineages: VIM-1 like, VIM-2 like, and VIM-7 like [2]. Among MBLs, VIM enzymes are commonly detected in European/Mediterranean countries such as Italy and Greece. In particular, VIM-1 and VIM-4 are geographically predominant in Europe, VIM-2 is globally distributed in *P. aeruginosa*, VIM-3 is widespread in Taiwan, VIM-6 in Asia, and VIM-7 in USA [3]. The *bla*_VIM-type_ genes are often inserted within class 1 integrons and located on plasmids playing a key role in the interspecies distribution of such determinants [4]. According to Matsumura et al. (2017), more than 20 different VIM-harboring integrons have been described [5]. Geographically, In*87*, In*624*, and In*916* are spread mostly in European countries and associated with *bla*_VIM-1_; In*339* is globally distributed and harbors *bla*_VIM-2_; In*450* in Taiwan carrying *bla*_VIM-3_; and In*496*, harboring *bla*_VIM-6_, circulates across the Asian continent. Moreover, In*916* has been identified in *Escherichia coli* (*E. coli*), *Klebsiella pneumoniae* (*K. pneumoniae*), *Enterobacter cloacae* (*E. cloacae*), *Klebsiella aerogenes* (*K. aerogenes*), and *Klebsiella oxytoca* (*K. oxytoca*); In*339* has been reported in *P. aeruginosa* and *Acinetobacter baumannii* (*A. baumannii*); and In*496* in *P. aeruginosa* and *Pseudomonas putida* (*P. putida*) [5]. The *bla*_VIM_ genes are mainly reported in IncN [6,7], IncY [8], IncR [9], and IncA type plasmids [10]. The IncA group, once assigned to the IncA/C family, was later allocated to a separate incompatibility group, not yet well characterized and recently recognized as an alternative reservoir of carbapenemase genes in the *Enterobacterales* family [11,12]. Here we characterize a VIM-producing IncA plasmid isolated from a clinical ST69 *E. coli* strain from an Italian Long-Term Care Facility (LTCF) inpatient.

## 2. Materials and Methods

### 2.1. Case Presentation, Antimicrobial Susceptibility Test and Molecular Investigations

On the 2nd of May 2018, a rectal swab was collected from a female patient, resident in the GP1 ward of the rehabilitation center “Giovanni Paolo II” (Milan, Italy). The swab, collected as part of a carbapenemase passive surveillance screening, was sent to the Clinical Microbiology Laboratory of the ASP “Golgi-Redaelli” of Milan for initial phenotypic characterization. Preliminary investigation, through Vitek-2 (bioMérieux, Marcy-l’Étoile, France) and a phenotypic synergy test, revealed the presence of a carbapenemase-producing *E. coli* strain. The isolate was sent to the Microbiology Laboratory of University of Pavia and to the “Biomedical Center” in Plzen, Czech Republic, for further molecular investigation. The species identification was confirmed through MALDI-TOF MS (Matrix-Assisted Laser Desorption Ionization-Time of Flight Mass Spectrometry) using MALDI Biotyper software (Brucker Daltonics, Bremen, Germany). Carbapenemase production was confirmed by meropenem hydrolysis assay [13], and antimicrobial susceptibility profiles were obtained by Microscan AutoScan-4 (Beckman-Coulter, Brea, CA, USA) and interpreted in accordance with EUCAST 2020 clinical breakpoints (https://www.eucast.org/fileadmin/src/media/PDFs/EUCAST_files/Breakpoint_tables/v_10.0_Breakpoint_Tables.pdf). Fosfomycin, colistin, and meropenem minimum inhibitory concentrations (MICs) were confirmed through broth-microdilution. Production of class B, D, and A carbapenemases was evaluated using disk combination synergy tests with meropenem and EDTA, temocillin, and phenylboronic acid, as inhibitors [14,15,16], respectively. The presence of carbapenemase genes was confirmed by polymerase chain reaction (PCR) as described by Shirani et al. 2016 [17]. Phylogenetic groups were determined by a two-step triplex PCR as described by Clermont et al. 2000 [18].

### 2.2. Conjugation Assay

To test the transferability of the *bla*_VIM-type_ gene, conjugation experiments were performed in Mueller Hilton (MH) broth (OXOID, Basingstoke, UK) using *E. coli* A15^r^Rif as the recipient. Transconjugant selection was assessed on MH agar (OXOID, Basingstoke, UK) plates supplemented with rifampicin (100 mg/μL) (Sigma-Aldrich, St. Louis, MO, USA) and ampicillin (50 mg/μL) (Sigma-Aldrich, St. Louis, MO, USA). The presence of the *bla*_VIM-type_ gene in the transconjugants was confirmed through PCR.

### 2.3. Whole-Genome Sequencing (WGS)

The *E. coli* strain designated as 550 was subjected to whole genome DNA extraction using the NucleoSpin Microbial DNA kit (Macherey-Nagel, Duren, Germany). The obtained DNA was sheared using Megaruptor 2 using the Hydropore-long (Diagenode). Library preparation of the sheared DNA was performed in accordance with the manufacturer’s recommendation for microbial multiplexing for the Express kit 2.0 (Pacific Biosciences, Menlo Park, CA, USA). No size selection was performed during library preparation. The constructed library was sequenced using long-reads sequencing technology on Sequel I (Pacific Biosciences, Menlo Park, CA, USA).

### 2.4. Whole-Genome-Sequencing-Data Analysis

Genome assembly was performed with minimum seed coverage of 30×, using the “Microbial Assembly” pipeline offered by “SMRT Link v8.0”. Antibiotic resistance genes, plasmid replicons, virulence factors, serotype, FimH type, FumC type, MLST, pMLST, and phage detection was obtained through uploading assembled contigs to ResFinder (https://cge.cbs.dtu.dk/services/ResFinder/) [19], PlasmidFinder (https://cge.cbs.dtu.dk/services/PlasmidFinder/) [20], VirulenceFinder (https://cge.cbs.dtu.dk/services/VirulenceFinder/) [21], VFDB: Virulence factors database (http://www.mgc.ac.cn/VFs/) [22], SeroTypeFinder 2.0 (https://cge.cbs.dtu.dk/services/SerotypeFinder/) [23], FimTyper 1.0 (https://cge.cbs.dtu.dk/services/FimTyper/) [24], CHTyper 1.0 (https://cge.cbs.dtu.dk/services/chtyper/) [25], MLST 2.0 (https://cge.cbs.dtu.dk/services/MLST/) [26], pMLST 2.0 (https://cge.cbs.dtu.dk/services/pMLST/) [20], and Phaster (https://phaster.ca) [27]. The genome was annotated by the NCBI Prokaryotic Genome Annotation Pipeline (PGAP). Plasmid comparisons were assessed through the Blast Ring Image Generator (BRIG) application (http://brig.sourceforge.net) and EasyFig (https://mjsull.github.io/Easyfig/) [28].

### 2.5. Nucleotide Accession Numbers

The chromosome sequence of 550 and the plasmid sequences of p550_IncA_VIM_1 and p550_IncB_O_K_Z were deposited in GenBank under the accession numbers CP058223, CP058224, and CP058225, respectively.

## 3. Results

The strain showed a multi-drug resistance (MDR) profile, expressing resistance against ampicillin, piperacillin, 3rd and 4th generation cephalosporins, ertapenem, gentamycin, tobramycin, and trimethoprim-sulfamethoxazole, yet remained susceptible to amikacin, colistin, fosfomycin, meropenem, imipenem, ciprofloxacin, and tigecycline (Table 1). The *E. coli* 550 strain was identified as an MBL producer by disk combination synergy test, and as *bla*_VIM-1_ positive through PCR and sequencing. A conjugation assay confirmed the transferability of the plasmid carrying the carbapenemase gene (Table 1).

Whole-genome sequencing revealed the presence of three contigs: a complete circular chromosome with a size of 4,962,700 bp, and two complete circular contigs corresponding to two different plasmids of 162,608 bp and 100,306 bp in size. The isolate belonged to the serotype O15:H18, uropathogenic sequence type ST69 (Achtman scheme), phylogenetic group D and CH-Type FumC35/FimH27 (fimbrial adhesion gene *fimH* with allele 27 and fumarate hydratase class II gene *fumC* with allele 35). Moreover, the strain’s chromosome harbored several virulence genes involved in: adhesion (hemorragic *E. coli* pilus, *EaeH*, Type I fimbriae), autotransporter (*ag43*: autoaggregation and flocculation of *E. coli* cells in static cultures [29], *cah*: carbonic anhydrase, *air:* enteroaggregative immunoglobulin repeat protein, *vat*: vacuolating autotransporter gene which contributes to uropathogenic *E. coli* (UPEC) fitness during systemic infection, invasion (*ibeB* and *ibeC* facilitates invasion of brain endothelial cells), iron uptake (*chu*: Hemin uptake, *sit*: Iron/manganese transport and Yersiniabactin siderophore), secretion (Type III secretion system), and toxin production (*clyA*: expressing the pore-forming hemolytic and cytotoxic cytolysin A). Additionally, the chromosome harbored an antibiotic resistance gene (*mdf(A)*) coding for macrolides resistance and two phages free of antibiotic resistance genes (Table 2).

**Figure 1:**
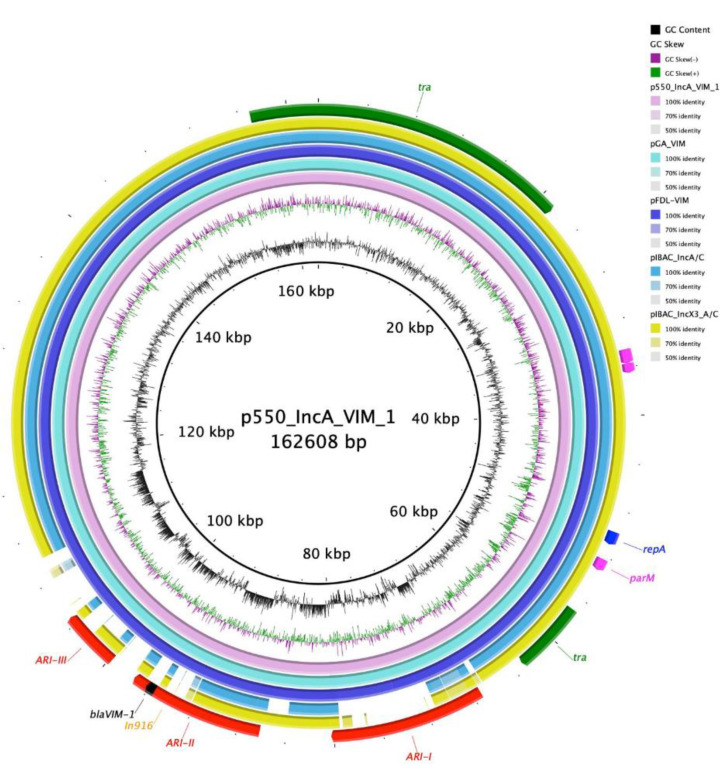
Circular map of p550_IncA_VIM_1 against pGA_VIM (pink), pFDL-VIM (turquoise), pIBAC_IncA/C (violet), and pIBAC_Incx3_A/C (yellow). At the outer curved segments; red, yellow, black, green, purple and blue corresponds to ARIs, In*916*, *bla*_VIM-1_, *tra* region, maintenance and stability region, and *repA*.

The second contig belonged to an IncA plasmid (p550_IncA_VIM_1: 162,608 bp) and pMLST IncA/C 12. The plasmid harbored genes coding for aminoglycoside resistance (*aac(6’)-Ib4*, *ant(3″)-Ia*, *aph(3″)-Ib*, *aph(3’)-XV*, *aph(6)-Id*), β-lactam resistance (*bla*_SHV-12_, *bla*_VIM-1_), macrolides resistance (*mph(A)*), phenicol resistance (*catB2*), quinolones resistance (*qnrS1*), sulphonamide resistance (*sul1*, *sul2*), and trimethoprim resistance (*dfrA14*) genes. p550_IncA_VIM_1 shared high identity scores with the pGA_VIM plasmid (182,016 bp) collected from a clinical ST12 *E. coli* strain in Italy (MN783743.2 [10]; 100% sequence and identity), pFDL-VIM plasmid (164,903 bp) isolated from a clinical *K. oxytoca* in Italy (MN783744.1 [10]; sequence coverage 100%, sequence identity 99.99%), pIBAC_IncA/C plasmid (145,294 bp) isolated from a clinical *Citrobacter freundii* (*C. freundii)* isolated in Italy (MH594477.1 [11]; 84% sequence coverage, 99.99% sequence identity), and pIBAC_Incx3_A/C plasmid (192,802 bp) isolated from a clinical *C. freundii* in Italy (MH594478.1 [11]; 87% sequence coverage and 100% sequence identity) (Figure 1). p550_IncA_VIM_1 backbone harbored conjugal transfer genes (*tra*), a replication initiation gene (*repA*), maintenance and stability genes (*parA*, *parM*), genes coding for mercuric uptake system (*mer*), and toxin/antitoxin system (*HigB/HipA*). Moreover, the plasmid contained three antimicrobial resistance islands (ARIs); ARI-I (12,914 bp) flanked by IS*26* on both ends in opposite orientation and harbored *sul-2*, *aph(6)-Id* and *qnrS1* genes. When blasted, ARI-I showed perfect identity with pKC-BO-N1-VIM found in a *Kluyvera cryocrescens* collected from a rectal swab from Italy (MG228427.1; query cover 100%, identity 100%). ARI-II (11,263 bp), flanked by an IS*4321R* and an *IntI1* in the same orientation, and harbored an In*916* integron with a cassette containing *sul2*, *catB2*, *ant(3″)-Ia, aph(3′)-XV*, *aac(6′)-Ib4* and *bla*VIM-1 genes. When blasted, the region shared high identity score with pKC-BO-N1-VIM (MG228427.1; query cover 100%, identity 100%). Moreover, a mercury uptake system region separated the two ARIs. ARI-III, of 4895 bp, contained just the *bla*_SHV-12_ gene, flanked by an IS*6-like* element and an IS*26* in opposite orientation. This region revealed high identities with pFDL-VIM found in *K. oxytoca* from Italy (MN783744.1; query cover 100%, identity 100%).

**Figure 2:**
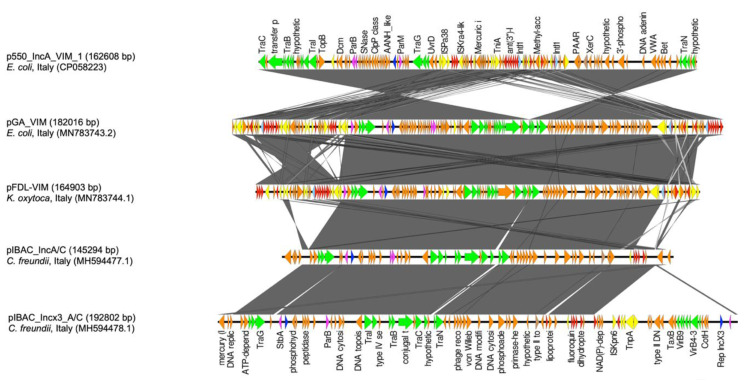
Genetic linear map of p550_IncA_VIM_1, pGA_VIM, pFDL-VIM, pIBAC_IncA/C, and pIBAC_Incx3_A/C. Replicons, partitioning genes, mobile elements, conjugal transfer genes, antibiotic resistance, and other remaining genes are designated by blue, purple, yellow, green, red, and orange, respectively. Gray shaded area shows nucleotide similarity.

p550_IncA_VIM_1 shared most of the IncA backbone with pGA_VIM, pFDL-VIM, pIBAC_IncA/C, and pIBAC_Incx3_A/C. Nevertheless, regarding ARIs, p550_IncA_VIM_1 and pGA_VIM shared most of the region; both plasmids shared the ARI-I region but in the opposite direction. Conversely, pGA_VIM harbored two copies of ARI-II while sharing one copy with p550_IncA_VIM_1 but in the opposite orientation. Moreover, the ARI-III region was also shared by both plasmids in opposite directions except for the IS*26*. In pGA_VIM, the “mercury system region” directly flanked the first copy of the ARI-II region, whereas in p550_IncA_VIM_1 it separated ARI-I and ARI-II, maintaining the same orientation. The orientation of the mercury system region and the opposite orientation of ARI-II in p550_IncA_VIM_1 suggests a recombination event responsible for the genetic restructuring of this region (Figure 2).

The third contig belonged to an IncK/Z plasmid (p550_Inc_B_O_K_Z; 100306 bp) and did not harbor any antibiotic resistance gene.

## 4. Discussion

Here, we report the first detection of a VIM-producing ST69 *E. coli* strain in Italy. *E. coli* ST69 is a member of the extraintestinal pathogenic *E. coli* (ExPEC) group, mostly involved in urinary tract infections (UTIs). It was first detected in 1999, in a study conducted in California, among 255 *E. coli* collected from urine of women with UTIs [30]. The ST69 lineage is part of the phylogenetic group D and belongs to the clonal group A (CGA) [31]. This group reported several serotypes such as O11, O15, O86, O125ab, and O25b [32]. ST69 has been associated with high virulence and pathogenicity, due to several virulence genes content coding for adhesins, toxins, autotransporters, and siderophores [33]. It has often been associated with trimethoprim-sulphametoxazole resistance and to the expression of CTX-M and TEM type enzymes. Recently, sporadic cases of ST69 *E. coli,* expressing several hydrolyzing enzymes, such as KPC-3 [34], NDM-1 [35] and mcr-1, have been reported [36]. Moreover, Fibke et al. identified a possible link between acquisition/infection with ST69 and travel histories. Additionally, the consumption of high-risk foods such as raw meat or vegetables, undercooked eggs, and seafood could play a role in the acquisition/infection with ST69 [37]. These data support the definition of ST69 as a high-risk clone, with increased ability for antimicrobial resistance genes acquisition. Moreover, the presence of type 1 fimbriae (*fimH27*), has been correlated with persisting colonization and bacteremia in patients [38].

VIM-type enzymes are widely detected in Italy and in Europe [39,40], harbored predominantly on IncN plasmids [6,7]. The expression of a VIM-1 enzyme by a high-risk ST69 clone, can represent a challenge, limiting the therapeutic options. In particular, the spread of VIM enzymes reduces the efficacy of the recently introduced therapeutic options such as ceftazidime-avibactam (CAZ-AVI). According to Arcari et al. the rise of MBL-producer circulation could be facilitated by extending the CAZ-AVI usage for treatment of KPC-producing-strains [10].

IncA plasmids are highly conjugative plasmids which are not yet well characterized. Recently, studies have suggested that IncA plasmids could act as carbapenemase reservoirs in different *Enterobacterales* species [6,11,41]. Few reports of complete closed IncA plasmids have been reported in the NCBI database. Nevertheless, from the plasmids discussed in Figure 2, IncA plasmids maintained a relatively stable backbone. The aforementioned ability could be explained by the presence of different integrative hotspot regions in the IncA plasmid backbone, particularly IS*26*, IS*6*, and *IntI1* elements, as described by Johnson et al. 2012 [42].

In conclusion, the high identity with other IncA plasmids highlights the predisposition of the IncA group to acquire several antimicrobial resistance genes. These data emphasize the overall important role of IncA plasmids in disseminating carbapenemase genes and, in particular, the *bla*_VIM-1_ in Italy [10]. The ability of these plasmids to accumulate different antibiotic resistant determinants in a high-risk clone, poses a health threat that might be difficult to control.

## Figures and Tables

**Table 1:** Antimicrobial susceptibility profile of *E. coli* A15, *E. coli* 550, and the transconjugant A15*E. coli 550 MIC (minimum inhibitory concentration, mg/L).

	AMP	AMS	ATM	CTX	FEP	CAZ	CIP	CO	FOS	ETP	MP	IMP	CN	TOB	TGC	TMX
***E. coli*** **A15**	2 S	2 S	≤0.125 S	≤0.068 S	≤0.125 S	≤0.25 S	≤0.068 S	≤0.5 S	≤0.125 S	≤0.032 S	≤0.125 S	≤0.125 S	≤0.25 S	≤0.125 S	≤0.125 S	≤0.068 S
***E. coli*** **550**	>128 R	>128 R	16 R	>8 R	16 R	>16 R	0.25 S	0.25 S	8 S	1 R	1 S	2 S	1 S	4 R	0.25 S	>4 R
A15* ***E. coli*** **550**	128 R	128 R	16 R	>8 R	8 R	>16 R	0.25 S	0.25 S	8 S	0.38 S	1 S	2 S	0.5 S	2 S	0.12 5S	>4 R

MIC, minimum inhibitory concentration: ampicillin; AMP, ampicillin/sulbactam; AMS, aztreonam; ATM, cefotaxime; CTX, cefepime; FEP, ceftazidime; CAZ, ciprofloxacin; CIP, colistin; CO, fosfomycin; FOS, ertapenem; ETP, meropenem; MP, imipenem; IMP, gentamycin; CN, tobramycin; TO, tigecycline; TIG; TMX, trimethoprim/sulfamethoxazole. R-resistant, S-susceptible.

**Table 2:** Antibiotic resistance genes and virulence determinants detected on the chromosome and the plasmid of the isolate 550.

Position	Antibiotic Resistance	Adhesion	Autotransporter	Invasion	Iron Uptake	Secretion	Toxin
**Chromosome**	*mdf(A)*	Pilus, *EaeH*, Type I fimbriae	*ag43, cah, air, vat*	*ibeB, ibeC*	*chu, sit*	Type III Secretion System	*clyA*
**p550_IncA_VIM_1**	*aac(6′)-lb4, ant(3″)-Ia, aph(3″)-lb, aph(3′)-XV, aph(6)-ld, bla*_SHV-12_ *, bla*_VIM-1_ *, mph(A), catB2, qnrS1, sul1, sul2, dfrA14*						
